# Transparency and Representation in Clinical Research Utilizing Artificial Intelligence in Oncology: A Scoping Review

**DOI:** 10.1002/cam4.70728

**Published:** 2025-03-09

**Authors:** Anjali J. D'Amiano, Tia Cheunkarndee, Chinenye Azoba, Krista Y. Chen, Raymond H. Mak, Subha Perni

**Affiliations:** ^1^ Johns Hopkins University School of Medicine Baltimore Maryland USA; ^2^ Brigham and Women's Hospital/Dana‐Farber Cancer Institute Harvard Medical School Boston Massachusetts USA; ^3^ The University of Texas MD Anderson Cancer Center Houston Texas USA

## Abstract

**Introduction:**

Artificial intelligence (AI) has significant potential to improve health outcomes in oncology. However, as AI utility increases, it is imperative to ensure that these models do not systematize racial and ethnic bias and further perpetuate disparities in health. This scoping review evaluates the transparency of demographic data reporting and diversity of participants included in published clinical studies utilizing AI in oncology.

**Methods:**

We utilized PubMed to search for peer‐reviewed research articles published between 2016 and 2021 with the query type “(“deep learning” or “machine learning” or “neural network” or “artificial intelligence”) and (“neoplas$” or “cancer$” or “tumor$” or “tumour$”).” We included clinical trials and original research studies and excluded reviews and meta‐analyses. Oncology‐related studies that described data sets used in training or validation of the AI models were eligible. Data regarding public reporting of patient demographics were collected, including age, sex at birth, and race. We used descriptive statistics to analyze these data across studies.

**Results:**

Out of 220 total studies, 118 were eligible and 47 (40%) had at least one described training or validation data set publicly available. 69 studies (58%) reported age data for patients included in training or validation sets, 60 studies (51%) reported sex, and six studies (5%) reported race. Of the studies that reported race, a range of 70.7%–93.4% of individuals were White. Only three studies reported racial demographic data with greater than two categories (i.e. “White” vs. “non‐White” or “White” vs. “Black”).

**Conclusions:**

We found that a minority of studies (5%) analyzed reported racial and ethnic demographic data. Furthermore, studies that did report racial demographic data had few non‐White patients. Increased transparency regarding reporting of demographics and greater representation in data sets is essential to ensure fair and unbiased clinical integration of AI in oncology.

## Graphical abstract

A minority of oncologic studies utilizing AI analyzed in this review reported the racial and ethnic demographic data of participants included in their training and validation sets. In studies that did report racial demographic data, few non‐white patients were included in the datasets.

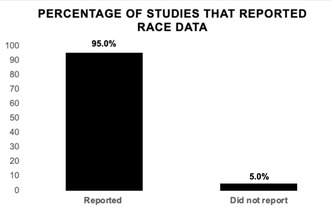

## 1 Introduction

Artificial intelligence (AI) algorithms, which broadly include those implementing machine learning, neural networks, and/or deep learning methods, have great potential to revolutionize oncology [1, 2]. Recent years have seen a substantial rise in research, implementation, and marketing for AI applications across the cancer care continuum, including screening, diagnosis, prognostication, patient decision support, treatment, and surveillance [1, 2]. For example, these advances range from enhanced methods of imaging and pathologic assessment for diagnosis to integration of genomics, biomarkers, and tumor profiling in predictive models for treatment response, as well as improved tumor segmentation and dose optimization in radiation therapy planning [3, 4, 5].

One crucial factor in the development of these tools is the utilization of high‐quality, representative datasets. The data used to train and validate a model directly influence its potential applications [6]. It is thus essential that datasets reflect a broad spectrum of real‐world scenarios. When datasets are flawed or lacking in diversity—algorithms learn to internalize these patterns as ground truth, reinforcing biases from the dataset into their predictions. Accordingly, various case studies in recent years have highlighted the potential of AI to reproduce and even amplify structural inequities and long‐standing disparities in healthcare [7, 8, 9, 10, 11, 12].

In response to this growing concern, international research consortiums such as the Consolidated Standards of Reporting Trials (CONSORT‐AI) and regulatory bodies such as the US Food and Drug Administration (FDA), which control entry of all medical devices into the US market, have developed frameworks and workgroups for the safe and effective development of AI in medicine [13, 14, 15]. Their guidelines have called for improved generalizability and validity of test datasets, increased transparency for users, collaboration with clinicians, and real‐world monitoring of post‐marketing performance [16, 17]. While absolute representation may be impossible to implement, a central mitigation strategy includes the characterization of datasets, calling on developers to make relevant information on the diversity of patient training sets and outcome measures available to the community of physicians who are responsible for interpreting information from these algorithms for clinical use so they may be aware of any potential biases.

As the use of AI rapidly increases in medicine, it is imperative to ensure that these models do not systematize racial and ethnic bias and further perpetuate disparities in health. Given these advancements in AI and well‐established cancer disparities based on minority and medically underserved statuses [18], we sought to investigate the role of AI models in propagating such disparities. The goals of this scoping review were twofold: first, to determine the transparency and availability of demographic information—predominantly race, as well as sex and age—in training and validation datasets used in AI models within published oncologic studies; and second, to evaluate the diversity of these datasets to identify potential performance biases and opportunities for improvement, with the aim of promoting more equitable health outcomes among cancer patients.

## 2 Methods

To evaluate the transparency of demographic data reporting and diversity of participants included in training and validation sets in published clinical studies utilizing AI in oncology, we utilized a scoping review approach to identify current literature in AI development. A scoping review approach was selected consistent with previous studies that have investigated the racial and ethnic disparities among AI algorithms used in clinical care [19]. Additionally, we utilized a scoping review to identify the publications utilizing AI models for oncology research, to determine the transparency of demographic data in training/validation sets, and to determine areas of potential bias and disparities [20].

**FIGURE 1:**
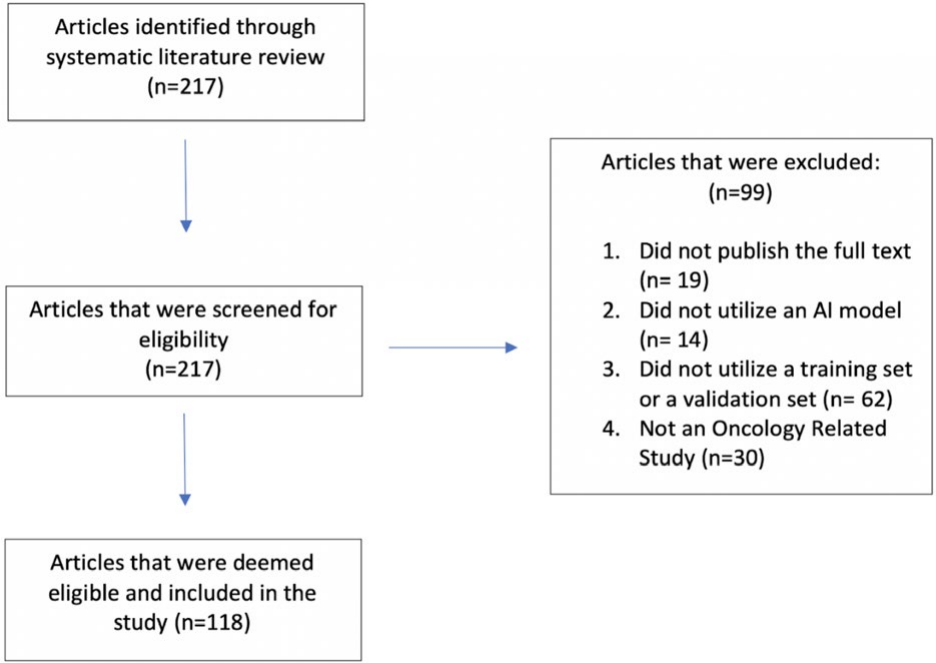
Inclusion and exclusion criteria to select eligible articles for the scoping review. Two hundred and seventeen articles were screened for eligibility, and 118 overall were eligible. Ninety‐nine studies were excluded for not publicly reporting the full text, no AI models, no training or validation sets in the methods, and not oncology‐related.

In this review, we used a PubMed extraction to search for peer‐reviewed research articles published between 2016 and 2021. Eligibility criteria included published trials of AI models for oncology research containing any age group and all cancer types, including solid and liquid tumors. To identify these articles, we searched the database with the query type “(“deep learning” or “machine learning” or “neural network” or “artificial intelligence”) AND (“neoplas$” or “cancer$” or “tumor$” or “tumor$”).” Oncology‐related article types in English including clinical trials, clinical studies, and randomized controlled trials were filtered for. In addition to these criteria, additional eligibility criteria included that the manuscript must have utilized either training sets and/or validation sets for the AI development in the main text. Furthermore, the papers must have presented AI models that had been internally or externally validated. We excluded reviews, systematic reviews, books, documents, and meta‐analyses. Additionally, we excluded abstract‐only publications. Overall, we identified 118 studies that met our criteria (Figure 1).

**FIGURE 2:**
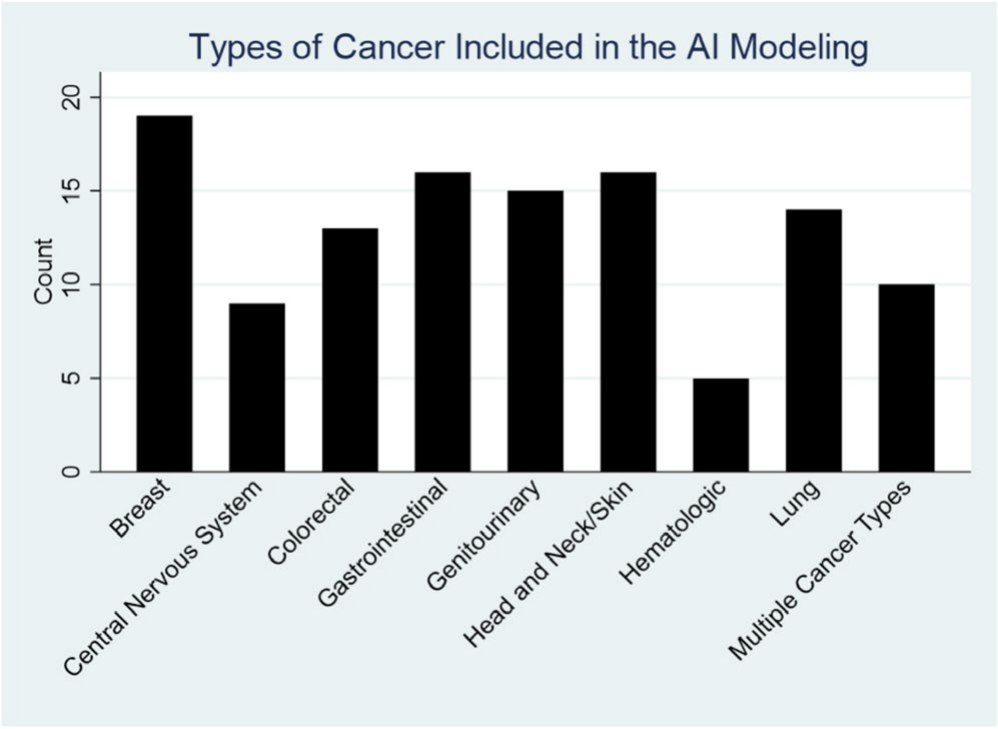
Domains of cancer included in the AI models. Breast cancer (*n* = 19) was the most represented in the studies analyzed in this scoping review, followed by gastrointestinal (*n* = 16), and head and neck/skin (*n* = 16). Hematologic (*n* = 4) cancer was the least represented.

Three investigators (AD, TC, CA) screened studies to determine if they met the above eligibility criteria and to ensure that the full text of these studies were available. The investigators then analyzed the domain of cancer type in each study and determined if the articles publicly reported the training and validating sets (Figure 2). If so, the number of training and validation sets that each study reported was collected. If a study reported multiple separate validation sets, then these datasets were also separated in our analysis.

The researchers also determined if the studies publicly reported patient demographics for the training and validating sets used to create the respective AI models, including age, sex at birth, and race. Age, sex at birth, and race were recorded for all patients in each training and validation set. Descriptive statistics, including the sample size, mean and median ages, weighted means of age, percent of women versus men, and percentages of race categories, were all calculated for each training and validation set for every study individually. Demographic data, including the mean and median ages, percent of women versus men, and percentages of race categories, were also calculated across studies.

## 3 Results

Out of 220 total studies, 118 were eligible and 47 had at least one described training or validation data set [21, 22, 23, 24, 25, 26, 27, 28, 29, 30, 31, 32, 33, 34, 35, 36, 37, 38, 39, 40, 41, 42, 43, 44, 45, 46, 47, 48, 49, 50, 51, 52, 53, 54, 55, 56, 57, 58, 59, 60, 61, 62, 63, 64, 65, 66, 67, 68, 69, 70, 71, 72, 73, 74, 75, 76, 77, 78, 79, 80, 81, 82, 83, 84, 85, 86, 87, 88, 89, 90, 91, 92, 93, 94, 95, 96, 97, 98, 99, 100, 101, 102, 103, 104, 105, 106, 107, 108, 109, 110, 111, 112, 113, 114, 115, 116, 117, 118, 119, 120, 121, 122, 123, 124, 125, 126, 127, 128, 129, 130, 131, 132]. All studies were published between 2016 and 2021. The main study characteristics, including cancer types, mean ages, and sex distribution, both overall and stratified by training and validation sets, are shown in (Table 1) and cancer types are shown in (Figure 2).

**TABLE 1:** Study characteristics. This table describes the publication dates, cancer types described, mean ages, and sex distribution in the articles that published their training/validation sets and/or reported age and sex distribution data. We describe this data for training sets, validation sets, and overall across all datasets.

	Training set	Validation sets	Overall
Years published	2016–2021	2016–2021	2016–2021
Cancer types (%)			
Breast	16.24	8.11	15.22
Central nervous system	7.69	8.11	0.00
Colorectal	11.11	18.92	17.39
Gastrointestinal	13.68	21.62	19.57
Genitourinary	12.82	10.81	10.87
Head and neck/skin	13.68	5.41	6.52
Hematologic	4.27	8.11	6.52
Lung	11.97	16.22	17.39
Multiple cancer types	8.55	2.70	6.52
Mean age (years)	56.6 ± 7.2	55.4 ± 11.7	56.8 ± 11.01
Sex distribution	49.1% Female 50.9% Male	54.2% Female 45.8% Male	51.5% Female 48.5% Male

Of these 118 eligible articles, 69 studies (58%) reported age data for patients who were included in training or validation sets. Across studies, age ranged from 16 days to 96 years For studies that reported mean age, the overall mean age of participants was 56.8 ± 11.0 years. The mean age of those used in the training sets was 56.6 ± 7.2 years, and the mean age of those used in the validation sets was 55.4 ± 11.7 years.

**FIGURE 3:**
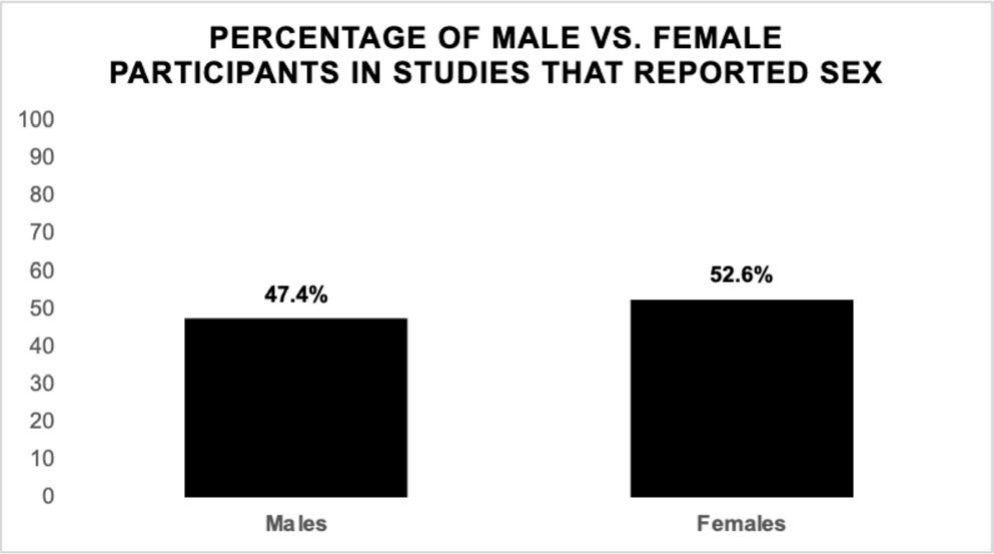
Male and female participants in studies that reported sex. Studies included 47.4% males and 52.6% females.

Additionally, 60 studies (51%) reported sex data for the participants. Across studies, 47.4% of patients included in studies with reported sex were male, and 52.6% were female (Figure 3). Thirty‐eight studies (63.3%) that reported sex included a majority of males, and 22 studies (36.7%) had a majority of females. Among the training sets, 49.1% of participants were female, and 50.9% were male. Regarding the validation sets, 54.2% of participants were female, and 45.8% were male.

**FIGURE 4:**
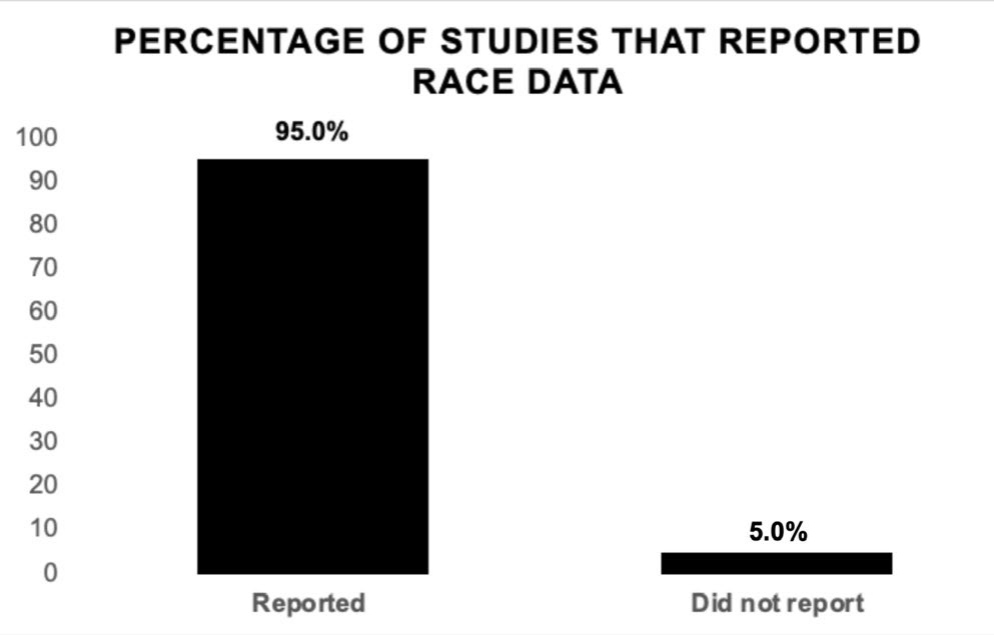
Percentages of studies that reported race. Out of 118 studies analyzed, six (5.0%) reported the racial demographics of the participants included in their training sets and/or validation sets, while 112 (95.0%) studies omitted this information.

**FIGURE 5:**
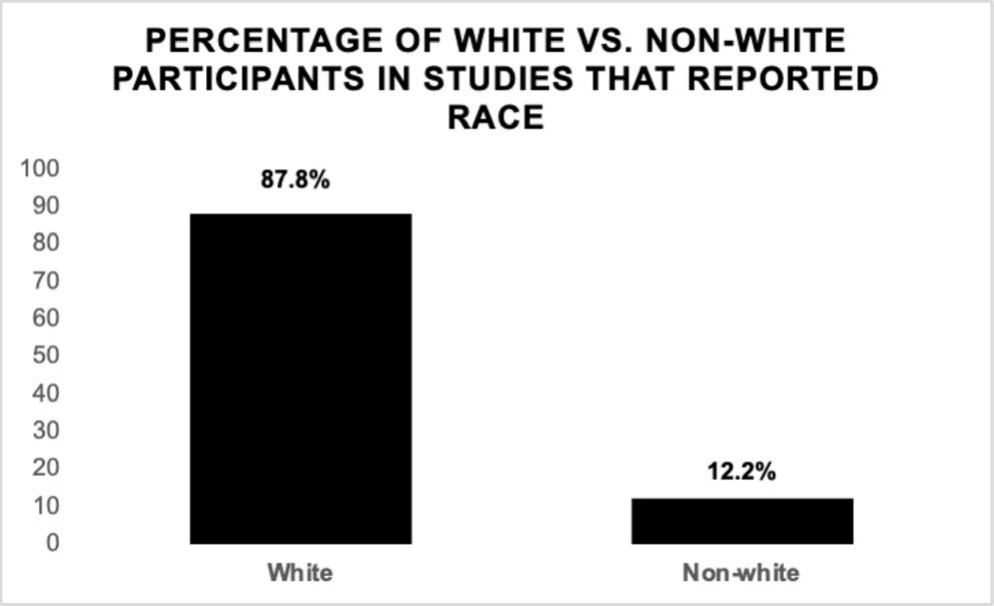
Percentage of white versus non‐white patients in studies that reported race. In the six studies that reported race, 87.8% of participants included in the training and/or validation sets were White while 12.2% were non‐White.

Among the 118 studies, only six studies (5.0%) reported patient race in at least one validation or training set, while 112 studies (95.0%) failed to report this data (Figure 4). We assessed these six studies, and a complete breakdown of racial demographics in the six studies that reported race is shown in (Table 2) [133, 134, 135, 136, 137, 138]. Within these studies that reported race, 70.7%–93.4% of individuals were White. Overall, 87.8% (*n* = 13,501) of participants included in the training or validation sets for these articles were White, while 12.2% (*n* = 1868) of participants were non‐White (Figure 5). The total counts in each racial category included 13,501 White, 1868 non‐White, 507 Black, 300 Hispanic, 92 Asian and 189 other patients. The overall median percentage of non‐white individuals across these studies was 14.4%.

**TABLE 2:** Breakdown of racial demographics in studies that reported race. In the six studies that reported racial demographics for their training and/or validation sets, 88% of participants (*n* = 13,501) were White while 12% (*n* = 1868) were non‐White. The total sum of each racial category included 13,501 White, 1868 non‐White, 507 Black, 300 Hispanic, 92 Asian, and 189 other patients. Out of these six studies, two (Study 1 and Study 6) utilized the categories White versus non‐White, while the other four utilized the categories White, Black, Hispanic, Asian, and other.

Source	Dai, et al. 2019 [138]	Gandelman, et al. 2019 [137]	Gates, et al. 2019 [136]	Hong, et al. 2020 [135]	Klein, et al. 2021 [134]	Lenchik, et al. 2021 [133]	Sum of each racial category
White	2949 (93.2%)	305 (90.0%)	17 (73.9%)	681 (70.7%)	3312 (81.2%)	6237 (91.7%)	13,501 (88%)
Non‐White	214 (6.8%)	34 (10.0%)	6 (26.1%)	282 (29.3%)	766 (18.8%)	566 (8.3%)	1868 (12%)
Black	—	7 (2.1%)	2 (8.7%)	220 (22.8%)	278 (6.8%)	—	507
Hispanic	—	0 (0.0%)	4 (17.4%)	0 (0.0%)	296 (7.3%)	—	300
Asian	—	17 (5.0%)	0 (0.0%)	0 (0.0%)	75 (1.8%)	—	92
Other	—	10 (2.9%)	0 (0.0%)	62 (6.4%)	117 (2.3%)	—	189

Regarding the non‐White racial category data, only four studies (3.4%) reported racial demographic data with more than two categories (i.e., “White” vs. “non‐White” or “White” vs. “Black”). Two studies [133, 138] utilized the categories White versus non‐White, while the other four utilized the categories White, Black, Hispanic, Asian, and other.

## 4 Discussion

As the influence of AI in medicine continues to grow, it is imperative to hold the application of these technologies to high standards to prevent negative impacts on patients and communities. To date, there is relatively little literature investigating avenues for bias in models used in healthcare and medicine; this scoping review aims to probe that question as it relates to studies in oncology. We analyzed 118 studies utilizing AI technology for machine‐based learning and neural networks in oncology to assess for transparency in the reporting of, and representation within, demographic data of training and validation sets. Of these, 47 studies (40%) had at least one training or validation set published and publicly available. With regard to the reporting of demographic information, while over half of all studies reported on the sex and/or age of the participants included in their training or validation sets (58% and 51%, respectively), we found that only six studies (5%) reported race. Of these six, four studies reported on racial demographic data utilizing specific racial categories rather than the binary categorizations of “White” versus “non‐White.” Across these six studies overall, 88% of patients included in the training and/or validation sets were White.

These results point to two primary needs that must be addressed in the utilization of AI in oncology. First, greater transparency in the reporting of this data is needed to assess representation in the training and validation of these models and further determine ways in which they may be perpetuating inequities against medically underserved populations. The implementation of clear guidelines and explicit requirements regarding these standards are needed to mitigate potential bias of AI technology in medicine. Required reporting of race, age, and sex characteristics among models is an important start to addressing these inequities. This is because studies with a lack of representation can be easily identified and potentially modified. However, it may not be fully sufficient to address disparities. This is because, as shown in these results, the studies that did report racial demographics utilized datasets that showed a general lack of diversity, indicating a need for more representative datasets. An increasing number of diverse training and validation sets is imperative to train machine learning and neural networks to become competent in caring for minority populations. Important repercussions for a lack of reporting include the propagation of racial disparities among health outcomes in cancer patients.

Given the ways in which structural inequities already impact cancer care, such as through barriers in access to care and patients of color presenting with later stages of disease at diagnosis [139], it is important to ensure that the rollout of AI technology does not embed the continuation of disparity into its algorithm. As such, AI models that are trained on primarily White patient datasets, such as those we identified in this study, are concerning for their lack of diversity. Without addressing this issue, structural inequities that are reflected in training and validation sets can all too easily manifest in the exacerbation of tangible health disparities among non‐White individuals.

There have been numerous accounts of AI model bias contributing to the systemization of structural inequities in healthcare. For example, one study found that a widely used algorithm gave Black patients the same risk score as White patients, despite the Black patients presenting as considerably sicker, leading to fewer resources being provided to them [7]. This bias was found to be due to the algorithm using healthcare cost as a proxy for health despite unequal access to care. This study highlighted the importance of label choice and how it is imperative to remain mindful of structural inequities the chosen labels may reflect as not to propagate these further.

Another study investigating algorithmic underdiagnosis in chest X‐ray pathology classification using three large, public datasets found consistent underdiagnosis of diseases in underserved patient populations, such as female patients, Black patients, Hispanic patients, younger patients, and patients of lower socioeconomic status [8]. This effect was compounded for intersectional underserved subpopulations. Such biases can lead to the perpetuation of delays in access to care.

On a broader scale, several other reviews investigating AI datasets have similarly found a lack of transparency and representation, indicating that this is a ubiquitous issue not limited to oncology. For example, a 2021 scoping review on AI use in dermatology by Daneshjou et al. reported a lack of dataset characterization and transparency, nonstandard and unverified disease labels, and an inability to assess patient diversity in the testing and development of the models used [19]. Furthermore, a 2022 systematic review on randomized clinical trials (RCTs) of ML interventions in healthcare by Plana et al. found that there was a high degree of variability in study adherence to CONSORT‐AI reporting standards, as well as variability in risk of bias and an overall lack of participants from underrepresented minority groups among studies that reported race and ethnicity data [140].

To date, there are a limited number of studies evaluating AI datasets in oncology. Oncology is a field driven by innovation—however, the issue of the lack of diversity in cancer clinical trials is a factor severely limiting the generalizability of trial results, particularly for African American and Hispanic patients [141]. AI is a novel, powerful technology that will likely significantly impact the future of oncology research and practice. As such, the implementation of AI in oncology necessitates diversity in datasets and transparency in demographic reporting. By providing insight into the existing body of literature on this topic, we hope to build awareness of important considerations for clinicians and researchers as the use of AI becomes more widespread.

It is imperative to center equity in the implementation of AI in healthcare and medicine. The training and validation sets used to generate these AI models can profoundly impact their performance; thus, these datasets must be robust and representative of diverse populations to avoid model bias and subsequent perpetuation of health disparities. While AI has the potential to have positive impacts across the spectrum of cancer care, its utilization must be approached cautiously and conscientiously. As discussed, model bias can have disastrous effects on patient care, especially if the algorithms or datasets used are reflective of structural inequities in healthcare. Based on our findings, many studies do not make their datasets publicly available, and a large majority do not report on the race of participants used in the training and validation of these models. Furthermore, among the studies that did report race, the datasets used were concerning for their lack of diversity. Implementing standardized guidelines to increase transparency in the reporting of the demographics of participants included in the training and validation of AI models, as well as ensuring representation among the datasets used in this process, are two important steps in addressing the pitfalls of AI utilization in medicine.

## 5 Limitations

As a scoping review, this study was limited by publications that were available on PubMed with the full text accessible. Additionally, many studies did not publish the training or validation sets, so we could not assess the demographic breakdown of these studies. Additionally, artificial intelligence is broadly defined here as encompassing machine learning, deep learning, and/or neural networks. However, this is consistent with scientific literature and industry discourse. This study does not explicitly include studies employing natural language processing. Furthermore, we focused our research on AI models used in implementing machine learning and neural networks for oncology‐related pursuits. These findings do not extend to other uses for AI, such as large language models.

## 6 Conclusion

This scoping review of AI‐based interventions utilized in oncology reveals two primary concerns: first, the lack of transparency in the reporting of racial demographics of patients included in the training and validation of these models and second, the issue of homogeneous datasets. Based on the few studies that reported racial demographics, datasets need to be more inclusive of non‐White patient populations. This lack of racial diversity is concerning for the potential perpetuation of racial and ethnic bias in these models. Instituting ubiquitous AI reporting guidelines for demographic reporting, as well as the utilization of datasets that are reflective of diverse populations, may ameliorate some of these concerns and promote greater inclusion in these studies.

## Author Contributions

**Anjali J. D'Amiano:** conceptualization (lead), data curation (lead), formal analysis (lead), investigation (lead), methodology (lead), project administration (lead), resources (lead), validation (lead), visualization (lead), writing – original draft (lead), writing – review and editing (lead). **Tia Cheunkarndee:** conceptualization (equal), data curation (equal), formal analysis (equal), investigation (equal), methodology (equal), resources (equal), supervision (equal), validation (equal), visualization (equal), writing – original draft (equal), writing – review and editing (supporting). **Chinenye Azoba:** conceptualization (equal), data curation (equal), investigation (equal). **Krista Y. Chen:** writing – original draft (supporting), writing – review and editing (supporting). **Raymond H. Mak:** supervision (equal), validation (equal), writing – review and editing (equal). **Subha Perni:** conceptualization (equal), data curation (equal), formal analysis (equal), funding acquisition (equal), project administration (lead), supervision (lead).

## Ethics Statement

This study adheres to the editorial policies and ethical considerations required by *Cancer Medicine*. This study does not include human studies and subjects, photographs with identifiable patients, or animal studies.

## Conflicts of Interest

The authors declare no conflicts of interest.

## Data Availability

The data that support the findings of this study are available from the corresponding author upon reasonable request.
